# New Electronic Health Records Screening Tools to Improve Detection of Emerging Psychosis

**DOI:** 10.3389/fpsyt.2021.698406

**Published:** 2021-07-14

**Authors:** Paolo Fusar-Poli

**Affiliations:** ^1^Department of Brain and Behavioral Sciences, University of Pavia, Pavia, Italy; ^2^Early Psychosis: Interventions and Clinical-Detection (EPIC) Lab, Department of Psychosis Studies, Institute of Psychiatry, Psychology & Neuroscience, King's College London, London, United Kingdom; ^3^OASIS Service, South London and Maudsley National Health Service Foundation Trust, London, United Kingdom

**Keywords:** psychosis, schizophrenia, electronic health record, prevention, machine-learning

A key rate-limiting step toward effective preventive approaches for psychotic disorders is the ability to detect most people who are at risk for developing this condition before they become acutely unwell (1). Improving detection of emerging psychosis requires an integrated approach to target secondary healthcare, primary care and the community (2). Precision psychiatry offers specific potential for improving detection of emerging psychosis (2). To specifically improve detection of emerging psychosis in secondary care, we have recently presented an innovative precision medicine approach, which leverages for the first-time artificial intelligence, dynamic prediction over time and electronic health records (EHRs): Dynamic ElecTronic hEalth reCord deTection (DETECT) (3). DETECT is based on a novel recurrent neural network model which predicts the risk of developing a first episode of psychosis over time. It employs demographics and medical events (in the categories diagnoses, prescriptions, procedures, encounters and admissions, observations, and laboratory test results) dynamically collected in the EHR as part of clinical routine (3). EHR data were obtained from IBM Explorys, which holds standardized, longitudinal, de-identified, patient-level EHR data pooled from different healthcare systems with distinct EHRs. A total of 102 030 individuals were randomly allocated to the development dataset, and the remaining 43 690 to the validation dataset (3). We demonstrated that DETECT's prognostic accuracy and AUROC was good: 0·787 and 0·868 in the development dataset and 0·774 and 0·856 in the validation dataset (3). We also produced prevalence-adjusted decision-curve analyses suggesting that DETECT was associated with a positive clinical net benefit in two different scenarios for detection of emerging psychosis (3).

We are submitting the current opinion piece to factually rectify several misleading statements raised by Cristea et al. (4) in their commentary to our manuscript “DETECT” (3) and stimulate constructive discussion.

First, the authors suggest that identifying individuals at risk of emerging psychosis is “inopportune” because of limited evidence for established preventive interventions (4). It is true that currently, there is no evidence to favor Cognitive Behavioral Therapy (CBT) over needs-based interventions for preventing psychosis (5–7). Beyond efficacy, the mechanisms of action of cognitive behavioral therapy remain unknown and non-specific, to the point that this intervention has been defined as a “black box” approach (8). However, absence of evidence is not evidence of absence. Current preventive CBT interventions may still be effective in subgroups of patients at risk for psychosis (9), calling for stratification and precision medicine approaches, such as DETECT (4). The authors' suggestion (4) of throwing out the baby with the bathwater and may impede future stratification studies as well as investigations of novel interventions.

It is also a misunderstanding to state that current interventions “might just delay and not prevent the onset of psychosis” (4) as something that speaks against their value. Delaying the onset of psychosis has value, both on the individual and societal level, because psychosis represents one of the most severe mental disorders. The authors (4) overlook that clinical services for individuals at risk of psychosis routinely offer an expanded package of care which includes comprehensive needs-based interventions focusing on psychosocial, vocational and familial necessities, along with public health initiatives such as outreach campaigns in collaboration with the local community to foster mental health literacy and promotion of good mental and physical health (10, 11). These efforts have important clinical benefits beyond prevention or delaying the onset of psychosis.

Second, the authors claim that only a minority of individuals at risk of psychosis “ultimately transition to the first episode of psychosis” (3). This claim overlooks the complex clinical needs of these individuals detailed above. Furthermore, this statement is conceptually misleading. The transition risk from a clinical high-risk state to the first onset of psychosis has recently been estimated at 25% at 3-years, which is about 50-fold higher than the general population (12). This risk is quantitative comparable with the probability of developing diabetes from a prediabetic stage, for which preventive interventions are under testing (13). More to this point, the authors (4) confuse the lack of transition to the first episode of psychosis with recovery and remission. The vast majority of adolescents and young adults at clinical high risk for psychosis who will not develop the disorder will still present persistent mental health problems at follow-up (9).

Third, the authors (4) raise the issue of economic costs associated with false positives and overdiagnosis (which is an incorrect terminology because the clinical at-risk status is an empirical research-based operationalization but not a diagnosis) but ignore the competing costs of false negatives, i.e., young people who will develop the most severe mental disorder and who will not receive potential beneficial preventive interventions. Furthermore, there are additional costs associated with persistent disability, as indicated above. Net benefits analyses accounting for both risk and harms showed potential value for screening using DETECT (4), with a 1-year real-world net benefit of €19,928 per person when early interventions for psychosis are implemented (14).

Fourth, we agree that preventive medicine in young people brings some ethical challenges in terms of the potential cost, inconvenience, social stigma and other harms of a false-positive designation in young people who might be at risk of psychosis. These concerns are corroborated by lack of valid biomarkers of risk (remarkably, there are no approved biomarkers in all of psychiatry). However, the authors (4) ignore that sharing an at-risk designation may not only be helpful but honors the ethical principle that young people have the right to know information relevant to their health. This is particularly relevant given the very real morbidity such as functional impairments, complex needs and persistent disability over time and beyond the risk of psychosis onset (15). We have demonstrated that ethically sensitive, automatic screening of electronic health records for emerging psychosis can be implemented prospectively in clinical practice, with high adherence of clinicians and positive endorsement of service users (16). More research is certainly needed to refine a solid ethical framework for implementing precision psychiatry and EHRs screening in a way that is acceptable to each cultural context.

Fifth, the authors raise concerns that individuals identified may be overexposed to antipsychotics. However, such treatment is discouraged by current preventive guidelines for young people at risk of psychosis (9). Therefore, antipsychotics are more likely to be inappropriately prescribed to young people at risk outside these preventive programmes (e.g., by their general practitioner or other healthcare professionals). Furthermore, psychological or psychosocial preventive interventions may also be associated with adverse effects, in particular in vulnerable groups. Similar interventions in humanitarian settings have been shown to worsen outcomes (17) and to be not more acceptable than the waiting-list condition (18).

Finally, we did not recommend using DETECT for clinical practice but clearly stated that further external validation is first needed. There is a dearth of implementation research in this field. In fact, a systematic review has found that only about 5% of the total pool of risk prediction models published in psychiatry is externally validated (most models may not cross the implementation threshold, as they would not improve outcomes) and that only 0.2% are being considered for implementation, highlighting a profound replication and translational gap (19). To overcome these caveats, future research should target refinements and replications of existing precision psychiatry algorithms and optimize their implementation (20).

We hope that DETECT will represent a starting point for future precision medicine studies that leverage the advancements of artificial intelligence and EHRs to improve detection of many young people at risk of psychosis to streamline the best evidence-based preventive care.

## Author Contributions

The author confirms being the sole contributor of this work and has approved it for publication.

## Conflict of Interest

The author declares that the research was conducted in the absence of any commercial or financial relationships that could be construed as a potential conflict of interest.
